# Soundness Conditions for Big-Step Semantics

**DOI:** 10.1007/978-3-030-44914-8_7

**Published:** 2020-04-18

**Authors:** Francesco Dagnino, Viviana Bono, Elena Zucca, Mariangiola Dezani-Ciancaglini

**Affiliations:** ETH Zurich, Zurich, Switzerland; 9 DIBRIS, University of Genova, Genoa, Italy; 10 Computer Science Department, University of Torino, Turin, Italy

## Abstract

We propose a general proof technique to show that a predicate is *sound*, that is, prevents stuck computation, *with respect to a big-step semantics*. This result may look surprising, since in big-step semantics there is no difference between non-terminating and stuck computations, hence soundness cannot even be *expressed*. The key idea is to define constructions yielding an extended version of a given arbitrary big-step semantics, where the difference is made explicit. The extended semantics are exploited in the meta-theory, notably they are necessary to show that the proof technique works. However, they remain *transparent* when using the proof technique, since it consists in checking three conditions on the original rules only, as we illustrate by several examples.

### 1 Introduction

The semantics of programming languages or software systems specifies, for each program/system configuration, its final result, if any. In the case of non-existence of a final result, there are two possibilities:

– either the computation stops with no final result, and there is no means to compute further: *stuck computation*,
– or the computation never stops: *non-termination*.

There are two main styles to define operationally a semantic relation: the *small-step* style [34,35], on top of a reduction relation representing single computation steps, or directly by a set of rules as in the *big-step* style [28]. Within a small-step semantics it is straightforward to make the distinction between stuck and non-terminating computations, while a typical drawback of the big-step style is that they are not distinguished (no judgement is derived in both cases).

For this reason, even though big-step semantics is generally more abstract, and sometimes more intuitive to design and therefore to debug and extend, in the literature much more effort has been devoted to study the meta-theory of small-step semantics, providing properties, and related proof techniques. Notably, the *soundness* of a type system (typing prevents stuck computation) can be proved by *progress* and *subject reduction* (also called *type preservation*) [40].

Our quest is then to provide a general proof technique to prove the soundness of a predicate with respect to an arbitrary big-step semantics. How can we achieve this result, given that in big-step formulation soundness cannot even be *expressed*, since non-termination is modelled as the absence of a final result exactly like stuck computation? The key idea is the following:

1. We define constructions *yielding an extended version of a given arbitrary big-step semantics*, where the difference between stuckness and non-termination is made explicit. In a sense, these constructions show that the distinction was "hidden" in the original semantics.
2. We provide a general proof technique by identifying *three sufficient conditions* on the original big-step rules to prove soundness.

Keypoint (2)'s three sufficient conditions are *local preservation*, *∃-progress*, and *∀-progress*. For *proving* the result that the three conditions actually ensure soundness, the setting up of the extended semantics from the given one is necessary, since otherwise, as said above, we could not even express the property.

*However, the three conditions deal only with the original rules of the given big-step semantics.* This means that, practically, in order to use the technique there is no need to deal with the extended semantics. This implies, in particular, that our approach does *not* increase the original number of rules. Moreover, the sufficient conditions are checked only on *single rules*, which makes explicit the proof fragments typically needed in a proof of soundness. Even though this is not exploited in this paper, this form of *locality* means *modularity*, in the sense that adding a new rule implies adding the corresponding proof fragment only.

As an important by-product, in order to formally define and prove correct the keypoints (1) and (2), we propose a formalisation of "what is a big-step semantics" which captures its essential features. Moreover, we support our approach by presenting several examples, demonstrating that: on the one hand, their soundness proof can be easily rephrased in terms of our technique, that is, by directly reasoning on big-step rules; on the other hand, our technique is essential when the property to be checked (for instance, the soundness of a type system) is *not preserved* by intermediate computation steps, whereas it holds for the final result. On a side note, our examples concern type systems, but the meta-theory we present in this work holds for any predicate.

We describe now in more detail the constructions of keypoint (1). Starting from an arbitrary big-step judgment $c \Rightarrow r$ that evaluates *configurations* $c$ into *results* $r$, the *first construction* produces an enriched judgement $c \Rightarrow_{\mathsf{tr}} t$ where $t$ is a *trace*, that is, the (finite or infinite) sequence of all the (sub)configurations encountered during the evaluation. In this way, by interpreting coinductively the rules of the extended semantics, an infinite trace models divergence (whereas no result corresponds to stuck computation). The *second construction* is in a sense dual. It is the *algorithmic* version of the well-known technique presented in Exercise 3.5.16 from the book [33] of adding a special result wrong explicitly modelling stuck computations (whereas no result corresponds to divergence).

By trace semantics and wrong semantics we can express two flavours of soundness, *soundness-may* and *soundness-must*, respectively, and show the correctness of the corresponding proof technique. This achieves our original aim, and it should be noted that *we define soundness with respect to a big-step semantics*

*within a big-step formulation*, without resorting to a small-step style (indeed, the two extended semantics are themselves big-step).

Lastly, we consider the issue of justifying on a formal basis that the two constructions are correct with respect to their expected meaning. For instance, for the wrong semantics we would like to be sure that *all* the cases are covered. To this end, we define a *third construction*, dubbed PEV for "partial evaluation", which makes explicit the *computations* of a big-step semantics, intended as the sequences of execution steps of the naturally associated evaluation algorithm. Formally, we obtain a reduction relation on approximated proof trees, so termination, non-termination and stuckness can be defined as usual. Then, the correctness of traces and wrong constructions is proved by showing they are equivalent to PEV for diverging and stuck computations, respectively.

In Sect. 2 we illustrate the meta-theory on a running example. In Sect. 3 we define the trace and wrong constructions. In Sect. 4 we express soundness in the *must* and *may* flavours, introduce the proof technique, and prove its correctness. In Sect. 5 we show in detail how to apply the technique to the running example, and other significant examples. In Sect. 6 we introduce the third construction and state that the three constructions are equivalent. Finally, in 7 and 8 we discuss related and further work and summarise our contribution. An extended version including an additional example, proofs omitted for lack of space, and technical details on the PEV semantics, can be found at http://arxiv.org/abs/2002.08738.

### 2 A meta-theory for big-step semantics

We introduce a formalisation of "what is a big-step semantics" that captures its essential features, subsuming a large class of examples (as testified in Sect. 5). This enables a general formal reasoning on an arbitrary big-step semantics.

A *big-step semantics* is a triple $\langle C, R, \mathcal{R}\rangle$ where:

- $C$ is a set of *configurations* $c$.
- $R \subseteq C$ is a set of *results* $r$. We define *judgments* $j \equiv c \Rightarrow r$, meaning that configuration $c$ evaluates to result $r$. Set $C(j) = c$ and $R(j) = r$.
- $\mathcal{R}$ is a set of *rules* $\rho$ of shape
  $$\frac{j_1 \;\cdots\; j_n \quad j_{n+1}}{c \Rightarrow R(j_{n+1})} \qquad \text{also written in } \textit{inline format}\text{: } \mathsf{rule}(j_1 \ldots j_n,\, j_{n+1},\, c)$$
  with $c \in C\backslash R$, where $j_1 \ldots j_n$ are the *dependencies* and $j_{n+1}$ is the *continuation*. Set $C(\rho){=}c$ and, for $i \in 1..n+1$, $C(\rho, i){=}C(j_i)$ and $R(\rho, i){=}R(j_i)$.
- For each result $r \in R$, we implicitly assume a single axiom $\dfrac{}{r \Rightarrow r}$. Hence, the only derivable judgment for $r$ is $r \Rightarrow r$, which we will call a *trivial* judgment.

We will use the inline format, more concise and manageable, for the development of the meta-theory, e.g., in constructions.

A rule corresponds to the following evaluation process for a non-result configuration: first, dependencies are evaluated in the given order, then the continuation is evaluated and its result is returned as result of the entire computation.

$e ::= x \mid v \mid e_1\ e_2 \mid \mathsf{succ}\, e \mid e_1 \oplus e_2$ expression
$v ::= n \mid \lambda x.e$ value

$$\text{(VAL)}\ \frac{}{v \Rightarrow v} \qquad \text{(APP)}\ \frac{e_1 \Rightarrow \lambda x.e \quad e_2 \Rightarrow v_2 \quad e[v_2/x] \Rightarrow v}{e_1\ e_2 \Rightarrow v} \qquad \text{(SUCC)}\ \frac{e \Rightarrow n}{\mathsf{succ}\ e \Rightarrow n+1}$$

$$\text{(CHOICE)}\ \frac{e_i \Rightarrow v}{e_1 \oplus e_2 \Rightarrow v}\ \ i = 1,2$$

(APP) $\mathsf{rule}(e_1 \Rightarrow \lambda x.e\ \ e_2 \Rightarrow v_2,\ e[v_2/x] \Rightarrow v,\ e_1\ e_2)$
(SUCC) $\mathsf{rule}(e \Rightarrow n,\ n+1 \Rightarrow n+1,\ \mathsf{succ}\, e)$
(CHOICE) $\mathsf{rule}(\epsilon,\ e_i \Rightarrow v,\ e_1 \oplus e_2)\ i = 1,2$

**Fig. 1.** Example of big-step semantics

Rules as defined above specify an inference system [1,30], whose inductive interpretation is, as usual, the semantic relation. However, they carry slightly more structure with respect to standard inference rules. Notably, premises are a sequence rather than a set, and the last premise plays a special role. Such additional structure does not affect the semantic relation defined by the rules, but allows abstract reasoning about an arbitrary big-step semantics, in particular it is relevant for defining the three constructions. In the following, we will write $\mathcal{R} \vdash c \Rightarrow r$ when the judgment $c \Rightarrow r$ is derivable in $\mathcal{R}$.

As customary, the (infinite) set of rules $\mathcal{R}$ is described by a finite set of meta-rules, each one with a finite number of premises. As a consequence, the number of premises of rules is not only finite but *bounded*. Since we have no notion of meta-rule, we model this feature (relevant in the following) as an explicit assumption:

BP there exists $b \in \mathbb{N}$ such that, for each $\rho \equiv \mathsf{rule}(j_1 \ldots j_n,\ j_{n+1},\ c)$, $n < b$.

We end this section illustrating the above definitions and conditions by a simple example: a $\lambda$-calculus with natural constants, successor and non-deterministic choice shown in Fig. 1. We present this example as an instance of our definition:

- Configurations and results are expressions, and values, respectively.[3]
- To have the set of (meta-)rules in our required shape, abbreviated in inline format in the bottom section of the figure:
  - axiom (VAL) can be omitted (it is implicitly assumed)
  - in (APP) we consider premises as a sequence rather than a set (the third premise is the continuation)
  - in (SUCC), which has no continuation, we add a dummy continuation
  - on the contrary, in (CHOICE) there is only the continuation (dependencies are the empty sequence, denoted $\epsilon$ in the inline format).

Note that (APP) corresponds to the standard left-to-right evaluation order. We could have chosen the right-to-left order instead:

$$\text{(APP-R)}\ \mathsf{rule}(e_2 \Rightarrow v_2\ \ e_1 \Rightarrow \lambda x.e\ ,\ e[v_2/x] \Rightarrow v,\ e_1\ e_2)$$

or even opt for a non-deterministic approach by taking both rules (APP) and

[3] In general, configurations may include additional components, see Sect. 5.2.

(APP-R). As said above, these different choices do not affect the semantic relation $c \Rightarrow r$ defined by the inference system, which is always the same. However, they will affect the way the extended semantics distinguishing stuck computation and non-termination is constructed. Indeed, if the evaluation of $e_1$ and $e_2$ is stuck and non-terminating, respectively, we should obtain stuck computation with rule (APP) and non-termination with rule (APP-R).

In summary, to see a typical big-step semantics as an instance of our definition, it is enough to assume an order (or more than one) on premises, make implicit the axiom for results, and add a dummy continuation when needed. In the examples (Sect. 5), we will assume a left-to-right order on premises, and omit dummy continuations to keep a more familiar style. In the technical part (Sect. 3, Sect. 4 and Sect. 6) we will adopt the inline format.

### 3 Extended semantics

In the following, we assume a big-step semantics $\langle C, R, \mathcal{R}\rangle$ and describe two constructions which make the distinction between non-termination and stuck computation explicit. In both cases, the approach is based on well-know ideas; the novel contribution is that, thanks to the meta-theory in Sect. 2, we provide a *general* construction working on an arbitrary big-step semantics.

#### 3.1 Traces

We denote by $C^\star$, $C^\omega$, and $C^\infty = C^\star \cup C^\omega$, respectively, the sets of finite, infinite, and possibly infinite *traces*, that is, sequences of configurations. We write $t \cdot t'$ for concatenation of $t \in C^\star$ with $t' \in C^\infty$.

We derive, from the judgement $c \Rightarrow r$, an enriched big-step judgement $c \Rightarrow_{\mathsf{tr}} t$ with $t \in C^\infty$. Intuitively, $t$ keeps trace of all the configurations visited during the evaluation, starting from $c$ itself. To define the trace semantics, we construct, starting from $\mathcal{R}$, a new set of rules $\mathcal{R}_{\mathsf{tr}}$, which are of two kinds:

**trace introduction** These rules enrich the standard semantics by finite traces: for each $\rho \equiv \mathsf{rule}(j_1 \ldots j_n,\, j_{n+1},\, c)$ in $\mathcal{R}$, and finite traces $t_1, \ldots, t_{n+1} \in C^\star$, we add the rule

$$\frac{C(j_1) \Rightarrow_{\mathsf{tr}} t_1 \cdot R(j_1) \quad \ldots \quad C(j_{n+1}) \Rightarrow_{\mathsf{tr}} t_{n+1} \cdot R(j_{n+1})}{c \Rightarrow_{\mathsf{tr}} c \cdot t_1 \cdot R(j_1) \cdot \ldots \cdot t_{n+1} \cdot R(j_{n+1})}$$

We denote this rule by $\mathsf{trace}(\rho, t_1, \ldots, t_{n+1})$, to highlight the relationship with the original rule $\rho$. We also add one axiom $\dfrac{}{r \Rightarrow_{\mathsf{tr}} r}$ for each result $r$.

Such rules derive judgements $c \Rightarrow t$ with $t \in C^\star$, for convergent computations.

**divergence propagation** These rules propagate divergence, that is, if a (sub)configuration in the premise of a rule diverges, then the subsequent premises are ignored and the configuration in the conclusion diverges as well: for each $\rho \equiv \mathsf{rule}(j_1 \ldots j_n,\, j_{n+1},\, c)$ in $\mathcal{R}$, index $i \in 1..n+1$, finite traces $t_1, \ldots, t_{i-1} \in C^\star$, and infinite trace $t$, we add the rule:

$$\frac{C(j_1) \Rightarrow_{\mathsf{tr}} t_1 \cdot R(j_1) \quad \ldots \quad C(j_{i-1}) \Rightarrow_{\mathsf{tr}} t_{i-1} \cdot R(j_{i-1}) \quad C(j_i) \Rightarrow t}{c \Rightarrow c \cdot t_1 \cdot R(j_1) \cdot \ldots \cdot t_{i-1} \cdot R(t_{i-1}) \cdot t}$$

$$\text{(APP-TRACE)}\ \frac{e_1 \Rightarrow_{\mathsf{tr}} t_1 \cdot \lambda x.e \quad e_2 \Rightarrow_{\mathsf{tr}} t_2 \cdot v_2 \quad e[v_2/x] \Rightarrow_{\mathsf{tr}} t \cdot v}{e_1\ e_2 \Rightarrow_{\mathsf{tr}} e_1\ e_2 \cdot t_1 \cdot \lambda x.e \cdot t_2 \cdot v_2 \cdot t \cdot v}\ t_1, t_2, t \in C^\star$$

$$\text{(DIV-APP-1)}\ \frac{e_1 \Rightarrow_{\mathsf{tr}} t}{e_1\ e_2 \Rightarrow_{\mathsf{tr}} e_1\ e_2 \cdot t}\ t \in C^\omega \qquad \text{(DIV-APP-2)}\ \frac{e_1 \Rightarrow_{\mathsf{tr}} t_1 \cdot \lambda x.e \quad e_2 \Rightarrow_{\mathsf{tr}} t}{e_1\ e_2 \Rightarrow_{\mathsf{tr}} e_1\ e_2 \cdot t_1 \cdot \lambda x.e \cdot t}\ t_1 \in C^\star, t \in C^\omega$$

$$\text{(DIV-APP-3)}\ \frac{e_1 \Rightarrow_{\mathsf{tr}} t_1 \cdot \lambda x.e \quad e_2 \Rightarrow_{\mathsf{tr}} t_2 \cdot v_2 \quad e[v_2/x] \Rightarrow_{\mathsf{tr}} t}{e_1\ e_2 \Rightarrow_{\mathsf{tr}} e_1\ e_2 \cdot t_1 \cdot \lambda x.e \cdot t_2 \cdot v_2 \cdot t}\ t_1, t_2 \in C^\star, t \in C^\omega$$

**Fig. 2.** Trace semantics for application

We denote this rule by $\mathsf{prop}(\rho, i, t_1, \ldots, t_{i-1}, t)$ to highlight the relationship with the original rule $\rho$. These rules derive judgements $c \Rightarrow_{\mathsf{tr}} t$ with $t \in C^\omega$, modelling diverging computations.

The inference system $\mathcal{R}_{\mathsf{tr}}$ must be interpreted *coinductively*, to properly model diverging computations. Indeed, since there is no axiom introducing an infinite trace, they can be derived only by an infinite proof tree. We write $\mathcal{R}_{\mathsf{tr}} \vdash c \Rightarrow_{\mathsf{tr}} t$ when the judgment $c \Rightarrow_{\mathsf{tr}} t$ is derivable in $\mathcal{R}_{\mathsf{tr}}$.

We show in Fig. 2 the rules obtained starting from meta-rule (APP) of the example (for other meta-rules the outcome is analogous).

For instance, set $\Omega = \omega\,\omega = (\lambda x.x\,x)\,(\lambda x.x\,x)$, and $t_\Omega$ the infinite trace $\Omega \cdot \omega \cdot \omega \cdot \Omega \cdot \omega \cdot \omega \cdot \ldots$, it is easy to see that the judgment $\Omega \Rightarrow_{\mathsf{tr}} t_\Omega$ can be derived by the following infinite tree:[4]

$$\text{(DIV-APP3)}\ \frac{\text{(TRACE-VAL)}\ \dfrac{}{\omega \Rightarrow_{\mathsf{tr}} \omega} \quad \text{(TRACE-VAL)}\ \dfrac{}{\omega \Rightarrow_{\mathsf{tr}} \omega} \quad \text{(DIV-APP3)}\ \dfrac{\vdots}{\omega\,\omega \equiv (x\,x)[\omega/x] \Rightarrow_{\mathsf{tr}} t_\Omega}}{\Omega \Rightarrow \Omega \cdot \omega \cdot \omega \cdot t_\Omega \equiv t_\Omega}$$

Note that *only* the judgment $\Omega \Rightarrow_{\mathsf{tr}} t_\Omega$ can be derived, that is, the trace semantics of $\Omega$ is uniquely determined to be $t_\Omega$, since the infinite proof tree forces the equation $t_\Omega = \Omega \cdot \omega\omega \cdot t_\Omega$. This example is a cyclic proof, but there are divergent computations with no circular derivation.

The trace construction is *conservative* with respect to the original semantics, that is, converging computations are not affected.

**Theorem 1.** *$\mathcal{R}_{\mathsf{tr}} \vdash c \Rightarrow_{\mathsf{tr}} t \cdot r$ for some $t \in C^\star$ iff $\mathcal{R} \vdash c \Rightarrow r$.*

#### 3.2 Wrong

A well-known technique [33] (Exercise 3.5.16) to distinguish between stuck and diverging computations, in a sense "dual" to the previous one, is to add a special result $\mathsf{wrong}$, so that $c \Rightarrow \mathsf{wrong}$ means that the evaluation of $c$ goes stuck.

In this case, to define an "automatic" version of the construction, starting from $\langle C, R, \mathcal{R}\rangle$, is a non-trivial problem. Our solution is based on defining a relation on rules, modelling *equality up to a certain index* $i$, also used for other aims

[4] To help the reader, we add equivalent expressions with a grey background.

in the following. Consider $\rho \equiv \mathsf{rule}(j_1 \ldots j_n,\ j_{n+1},\ c)$, $\rho' \equiv \mathsf{rule}(j'_1 \ldots j'_m,\ j'_{m+1},\ c')$, and an index $i \in 1..\min(n+1, m+1)$, then $\rho \sim_i \rho'$ if

– $c = c'$
– for all $k < i$, $j_k = j'_k$
– $C(j_i) = C(j'_i)$

Intuitively, this means that rules $\rho$ and $\rho'$ model the same computation until the $i$-th premise. Using this relation, we derive, from the judgment $c \Rightarrow r$, an enriched big-step judgement $c \Rightarrow r_{\mathsf{wr}}$ where $r_{\mathsf{wr}} \in R \cup \{\mathsf{wrong}\}$, defined by a set of rules $\mathcal{R}_{\mathsf{wr}}$ containing all rules in $\mathcal{R}$ and two other kinds of rules:

**wrong introduction** These rules derive wrong whenever the (sub)configuration in a premise of a rule reduces to a result which is not admitted in such (or any equivalent) rule: for each $\rho \equiv \mathsf{rule}(j_1 \ldots j_n,\ j_{n+1},\ c)$ in $\mathcal{R}$, index $i \in 1..n+1$, and result $r \in R$, if for all rules $\rho'$ such that $\rho \sim_i \rho'$, $R(\rho', i) \neq r$, then we add the rule $\mathsf{wrong}(\rho,\ i,\ r)$ as follows:

$$\frac{j_1 \ldots j_{i-1} \quad C(j_i) \Rightarrow r}{c \Rightarrow \mathsf{wrong}}$$

We also add an axiom $\dfrac{}{c \Rightarrow \mathsf{wrong}}$ for each configuration $c$ which is not the conclusion of any rule.

**wrong propagation** These rules propagate wrong analogously to those for divergence propagation: for each $\rho \equiv \mathsf{rule}(j_1 \ldots j_n,\ j_{n+1},\ c)$ in $\mathcal{R}$, and index $i \in 1..n+1$, we add the rule $\mathsf{prop}(\rho, i, \mathsf{wrong})$ as follows:

$$\frac{j_1 \ldots j_{i-1} \quad C(j_i) \Rightarrow \mathsf{wrong}}{c \Rightarrow \mathsf{wrong}}$$

We write $\mathcal{R}_{\mathsf{wr}} \vdash c \Rightarrow r_{\mathsf{wr}}$ when the judgment $c \Rightarrow r_{\mathsf{wr}}$ is derivable in $\mathcal{R}_{\mathsf{wr}}$.

We show in Fig. 3 the meta-rules for wrong introduction and propagation constructed starting from those for application and successor. For instance, rule (WRONG-APP) is introduced since in the original semantics there is rule (APP) with $e_1\ e_2$ in the consequence and $e_1$ in the first premise, but there is no equivalent rule (that is, with $e_1\ e_2$ in the consequence and $e_1$ in the first premise) such that the result in the first premise is $n$.

The wrong construction is conservative as well.

**Theorem 2.** *$\mathcal{R}_{\mathsf{wr}} \vdash c \Rightarrow r$ iff $\mathcal{R} \vdash c \Rightarrow r$.*

$$\text{(WRONG-APP)}\ \frac{e_1 \Rightarrow n}{e_1\ e_2 \Rightarrow \mathsf{wrong}} \qquad \text{(WRONG-SUCC)}\ \frac{e \Rightarrow \lambda x.e'}{\mathsf{succ}\ e \Rightarrow \mathsf{wrong}}$$

$$\text{(PROP-APP-1)}\ \frac{e_1 \Rightarrow \mathsf{wrong}}{e_1\ e_2 \Rightarrow \mathsf{wrong}} \qquad \text{(PROP-APP-2)}\ \frac{e_1 \Rightarrow \lambda x.e \quad e_2 \Rightarrow \mathsf{wrong}}{e_1\ e_2 \Rightarrow \mathsf{wrong}}$$

$$\text{(PROP-APP-3)}\ \frac{e_1 \Rightarrow \lambda x.e \quad e_2 \Rightarrow v_2 \quad e[v_2/x] \Rightarrow \mathsf{wrong}}{e_1\ e_2 \Rightarrow \mathsf{wrong}} \qquad \text{(PROP-SUCC)}\ \frac{e \Rightarrow \mathsf{wrong}}{\mathsf{succ}\ e \Rightarrow \mathsf{wrong}}$$

**Fig. 3.** Semantics with wrong for application and successor

## 4 Expressing and proving soundness

A predicate (for instance, a typing judgment) is *sound* when, informally, a program satisfying the predicate (e.g., a well-typed program) cannot *go wrong*, following Robin Milner's slogan [31]. In small-step style, as firstly formulated in [40], this is naturally expressed as follows: well-typed programs never reduce to terms which neither are values, nor can be further reduced (called *stuck* terms). The standard technique to ensure soundness is by subject reduction (well-typedness is preserved by reduction) and progress (a well-typed term is not stuck).

We discuss how soundness can be expressed for the two approaches previously presented and we introduce sufficient conditions. In other words, we provide a proof technique to show the soundness of a predicate with respect to a big-step semantics. As mentioned in the Introduction, the extended semantics is only needed to prove the correctness of the technique, whereas to *apply* the technique for a given big-step semantics it is enough to reason on the original rules.

### 4.1 Expressing soundness

In the following, we assume a big-step semantics $\langle C, R, \mathcal{R}\rangle$, and an *indexed predicate on configurations*, that is, a family $\Pi = (\Pi_\iota)_{\iota\in I}$, for $I$ set of *indexes*, with $\Pi_\iota \subseteq C$. A representative case is that, as in the examples of Sect. 5, the predicate is a typing judgment and the indexes are types; however, the proof technique could be applied to other kinds of predicates. When there is no ambiguity, we also denote by $\Pi$ the corresponding predicate $\bigcup_{\iota\in I} \Pi_\iota$ on $C$ (e.g., to be well-typed with an arbitrary type).

To discuss how to express soundness of $\Pi$, first of all note that, in the nondeterministic case (that is, there is possibly more than one computation for a configuration), we can distinguish two flavours of soundness [21]:

**soundness-must** (or simply soundness) no computation can be stuck
**soundness-may** at least one computation is not stuck

Soundness-must is the standard soundness in small-step semantics, and can be expressed in the wrong extension as follows:

**soundness-must (wrong)** If $c \in \Pi$, then $\mathcal{R}_{\mathsf{wr}} \not\vdash c \Rightarrow \mathsf{wrong}$

Instead, soundness-must *cannot* be expressed in the trace extension. Indeed, stuck computations are not explicitly modelled. Conversely, soundness-may can be expressed in the trace extension as follows:

**soundness-may (traces)** If $c \in \Pi$, then there is $t$ such that $\mathcal{R}_{\mathsf{tr}} \vdash c \Rightarrow_{\mathsf{tr}} t$

whereas cannot be expressed in the wrong semantics, since diverging computations are not modelled.

Of course soundness-must and soundness-may coincide in the deterministic case. Finally, note that indexes (e.g., the specific types of configurations) do not play any role in the above statements. However, they are relevant in the notion of *strong soundness*, introduced by [40]. Strong soundness holds if, for configurations satisfying $\Pi_\iota$ (e.g., having a given type), computation cannot be stuck, and moreover, produces a result satisfying $\Pi_\iota$ (e.g., of the same type) if terminating. Note that soundness alone does not even guarantee to obtain a result satisfying $\Pi$ (e.g., a well-typed result). The three conditions introduced in the following section actually ensure strong soundness.

In Sect. 4.2 we provide sufficient conditions for soundness-must, showing that they actually ensure soundness in the wrong semantics (Theorem 3). Then, in Sect. 4.3, we provide (weaker) sufficient conditions for soundness-may, and show that they actually ensure soundness-may in the trace semantics (Theorem 4).

#### 4.2 Conditions ensuring soundness-must

The three conditions which ensure the soundness-must property are *local preservation*, *∃-progress*, and ∀-progress. The names suggest that the former plays the role of the *type preservation (subject reduction)* property, and the latter two of the *progress* property in small-step semantics. However, as we will see, the correspondence is only rough, since the reasoning here is different.

Considering the first condition more closely, we use the name *preservation* rather than type preservation since, as already mentioned, the proof technique can be applied to arbitrary predicates. More importantly, *local* means that the condition is *on single rules* rather than on the semantic relation as a whole, as standard subject reduction. The same holds for the other two conditions.

**Definition 1 (S1: Local Preservation).** *For each* $\rho \equiv \mathsf{rule}(j_1 \ldots j_n,\ j_{n+1},\ c)$*, if* $c \in \Pi_\iota$*, then there exist* $\iota_1, \ldots, \iota_{n+1} \in I$*, with* $\iota_{n+1} = \iota$*, such that, for all* $k \in 1..n+1$*:*

$$\textit{if, for all } h < k,\ R(j_h) \in \Pi_{\iota_h},\ \textit{then } C(j_k) \in \Pi_{\iota_k}.$$

Thinking to the paradigmatic case where the indexes are types, for each rule $\rho$, if the configuration $c$ in the consequence has type $\iota$, we have to find types $\iota_1, \ldots, \iota_{n+1}$ which can be assigned to (the configurations in) the premises, in particular the same type as $c$ for the continuation. More precisely, we start finding type $\iota_1$, and successively find the type $\iota_k$ for (the configuration in) the $k$-th premise assuming that the results of all the previous premises have the expected types. Indeed, if all such previous premises are derivable, then the expected type should be preserved by their results; if some premise is not derivable, the considered rule is "useless". For instance, considering (an instantiation of) meta-rule (APP) $\mathsf{rule}(e_1 \Rightarrow \lambda x.e\ \ e_2 \Rightarrow v_2,\ e[v_2/x] \Rightarrow v,\ e_1\ e_2)$ in Sect. 2, we prove that $e[v_2/x]$ has the type $T$ of $e_1\ e_2$ under the assumption that $\lambda x.e$ has type $T' \to T$, and $v_2$ has type $T'$ (see the proof example in Sect. 5.1 for more details).
A counter-example to condition **S1** is discussed at the beginning of Sect. 5.3.

The following lemma states that local preservation actually implies *preservation* of the semantic relation as a whole.

**Lemma 1 (Preservation).** *Let* $\mathcal{R}$ *and* $\Pi$ *satisfy condition* **S1**. *If* $\mathcal{R} \vdash c \Rightarrow r$ *and* $c \in \Pi_\iota$*, then* $r \in \Pi_\iota$*.*

*Proof.* The proof is by a double induction. We denote by $RH$ and $IH$ the first and the second induction hypothesis, respectively. The first induction is on big-step rules. Axioms have conclusion $r \Rightarrow r$, hence the thesis holds since $r \in \Pi_\iota$ by hypothesis. Other rules have shape $\mathsf{rule}(j_1 \dots j_n,\, j_{n+1},\, c)$ with $c \in \Pi_\iota$. We prove by complete induction on $k \in 1..n+1$ that $C(j_k) \in \Pi_{\iota_k}$, for all $k \in 1..n+1$ and for some $\iota_1, \dots, \iota_{n+1} \in I$. By **S1**, there are $\iota_1, \dots, \iota_{n+1} \in I$ and $C(j_1) \in \Pi_{\iota_1}$. For $k > 1$, by $IH$ we know that $C(j_h) \in \Pi_{\iota_h}$, for all $h < k$. Then, by $RH$, we get that $R(j_h) \in \Pi_{\iota_h}$. Moreover by **S1**, $C(j_k) \in \Pi_{\iota_k}$, as needed. In particular, we have just proved that $C(j_{n+1}) \in \Pi_{\iota_{n+1}}$ and, since by **S1** $\iota_{n+1} = \iota$, we get $C(j_{n+1}) \in \Pi_\iota$. Then, by $RH$, we conclude that $r = R(j_{n+1}) \in \Pi_\iota$, as needed.

The following proposition is a form of local preservation where indexes (e.g., specific types) are not relevant, simpler to use in the proofs of Theorems 3 and 4.

**Proposition 1.** *Let $\mathcal{R}$ and $\Pi$ satisfy condition* **S1**. *For each $\mathsf{rule}(j_1 \dots j_n,\, j_{n+1},\, c)$ and $k \in 1..n+1$, if $c \in \Pi$ and, for all $h < k$, $\mathcal{R} \vdash j_h$, then $C(j_k) \in \Pi$.*

The second condition, named $\exists$-*progress*, ensures that, for configurations satisfying the predicate $\Pi$ (e.g., well-typed), we can *start constructing* a proof tree.

**Definition 2 (S2: $\exists$-progress).** *For each $c \in \Pi \backslash R$, $C(\rho) = c$ for some rule $\rho$.*

The third condition, named $\forall$-*progress*, ensures that, for configurations satisfying $\Pi$, we can *continue constructing* the proof tree. This condition uses the notion of rules *equivalent up-to an index* introduced at the beginning of Sect. 3.2.

**Definition 3 (S3: $\forall$-progress).** *For each $\rho \equiv \mathsf{rule}(j_1 \dots j_n,\, j_{n+1},\, c)$, if $c \in \Pi$, then, for each $k \in 1..n+1$:*

> *if, for all $h < k$, $\mathcal{R} \vdash j_h$ and $\mathcal{R} \vdash C(j_k) \Rightarrow r$, for some $r \in R$, then there is a rule $\rho' \sim_k \rho$ such that $R(\rho', k) = r$.*

We have to check, for each rule $\rho$, the following: if the configuration $c$ in the consequence satisfies the predicate (e.g., is well-typed), then, for each $k$, if the configuration in premise $k$ evaluates to some result $r$ (that is, $\mathcal{R} \vdash C(j_k) \Rightarrow r$), then there is a rule ($\rho$ itself or another rule with the same configuration in the consequence and the first $k-1$ premises) with such judgment as $k$-th premise. This check can be done under the assumption that all the previous premises are derivable. For instance, consider again (an instantiation of) the meta-rule (APP) $\mathsf{rule}(e_1 \Rightarrow \lambda x.e\ \ e_2 \Rightarrow v_2,\, e[v_2/x] \Rightarrow v,\, e_1\ e_2)$. Assuming that $e_1$ evaluates to some $v_1$, we have to check that there is a rule with first premise $e_1 \Rightarrow v_1$, in pratice, that $v_1$ is a $\lambda$-abstraction; in general, checking **S3** for a (meta-)rule amounts to show that (sub)configurations in the premises evaluate to results with the required shape (see also the proof example in Sect. 5.1).

*Soundness-must in* $\mathsf{wrong}$ *semantics* Recall that $\mathcal{R}_{\mathsf{wr}}$ is the extension of $\mathcal{R}$ with $\mathsf{wrong}$ (Sect. 3.2). We prove the claim of soundness-must with respect to $\mathcal{R}_{\mathsf{wr}}$.

**Theorem 3.** *Let $\mathcal{R}$ and $\Pi$ satisfy conditions* **S1**, **S2** *and* **S3**. *If $c \in \Pi$, then $\mathcal{R}_{\mathsf{wr}} \nvdash c \Rightarrow \mathsf{wrong}$.*

*Proof.* To prove the statement, we assume $\mathcal{R}_{\mathsf{wr}} \vdash c \Rightarrow \mathsf{wrong}$ and look for a contradiction. The proof is by induction on the derivation of $c \Rightarrow \mathsf{wrong}$.
If the last applied rule is an axiom, then, by construction, there is no rule $\rho \in \mathcal{R}$ such that $C(\rho) = c$, and this violates condition **S2**, since $c \in \Pi$.
If the last applied rule is $\mathsf{wrong}(\rho, i, r)$, with $\rho \equiv \mathsf{rule}(j_1 \ldots j_n, j_{n+1}, c)$, then, by hypothesis, for all $k < i$, $\mathcal{R}_{\mathsf{wr}} \vdash j_k$, and $\mathcal{R}_{\mathsf{wr}} \vdash C(j_i) \Rightarrow r$, and these judgments can also be derived in $\mathcal{R}$ by conservativity (Theorem 2). Furthermore, by construction of this rule, we know that there is no other rule $\rho' \sim_i \rho$ such that $R(\rho', i) = r$, and this violates condition **S3**, since $c \in \Pi$.
If the last applied rule is $\mathsf{prop}(\rho, i, \mathsf{wrong})$, with $\rho \equiv \mathsf{rule}(j_1 \ldots j_n, j_{n+1}, c)$, then, by hypothesis, for all $k < i$, $\mathcal{R}_{\mathsf{wr}} \vdash j_k$, and these judgments can also be derived in $\mathcal{R}$ by conservativity. Then, by Prop. 1 (which requires condition **S1**), since $c \in \Pi$, we have $C(j_i) \in \Pi$, hence we get the thesis by induction hypothesis.

Sect. 5.1 ends with examples not satisfying properties **S2** and **S3**.

#### 4.3 Conditions ensuring soundness-may

As discussed in Sect. 4.1, in the trace semantics we can only express a weaker form of soundness: at least one computation is not stuck (*soundness-may*). As the reader can expect, to ensure this property weaker sufficient conditions are enough: namely, condition **S1**, and another condition named *progress-may* and defined below.

We write $\mathcal{R} \nvdash c \Rightarrow$ if $c$ *does not converge* (there is no $r$ such that $\mathcal{R} \vdash c \Rightarrow r$).

**Definition 4 (S4: progress-may).** *For each $c \in \Pi \backslash R$, there is $\rho \equiv \mathsf{rule}(j_1 \ldots j_n, j_{n+1}, c)$ such that:*

> *if there is a (first) $k \in 1..n+1$ such that $\mathcal{R} \nvdash j_k$ and, for all $h < k$, $\mathcal{R} \vdash j_h$, then $\mathcal{R} \nvdash C(j_k) \Rightarrow$ .*

This condition can be informally understood as follows: we have to show that there is an either finite or infinite computation for $c$. If we find a rule where all premises are derivable (no $k$), then there is a finite computation. Otherwise, $c$ does not converge. In this case, we should find a rule where the configuration in the first non-derivable premise $k$ does not converge as well. Indeed, by coinductive reasoning (use of Lemma 2 below), we obtain that $c$ diverges. The following proposition states that this condition is indeed a weakening of **S2** and **S3**.

**Proposition 2.** *Conditions* **S2** *and* **S3** *imply condition* **S4**.

*Soundness-may in trace semantics* Recall that $\mathcal{R}_{\mathsf{tr}}$ is the extension of $\mathcal{R}$ with traces, defined in Sect. 3.1, where judgements have shape $c \Rightarrow_{\mathsf{tr}} t$, with $t \in C^{\infty}$.

The following lemma provides a proof principle useful to coinductively show that a property ensures the existence of an infinite trace, in particular to show Theorem 4. It is a slight variation of an analogous principle presented in [8].

**Lemma 2.** *Let $\mathcal{S} \subseteq C$ be a set. If, for all $c \in \mathcal{S}$, there are $\rho \equiv \mathsf{rule}(j_1 \dots j_n,\ j_{n+1},\ c)$ and $k \in 1..n+1$ such that*

1. *for all $h < k$, $\mathcal{R} \vdash j_h$, and*
2. *$C(j_k) \in \mathcal{S}$*

*then, for all $c \in \mathcal{S}$, there is $t \in C^\omega$ such that $\mathcal{R}_{\mathsf{tr}} \vdash c \Rightarrow_{\mathsf{tr}} t$.*

**Theorem 4.** *Let $\mathcal{R}$ and $\Pi$ satisfy conditions* **S1** *and* **S4**. *If $c \in \Pi$, then there is $t$ such that $\mathcal{R}_{\mathsf{tr}} \vdash c \Rightarrow_{\mathsf{tr}} t$.*

*Proof.* First note that, thanks to Theorem 1, the statement is equivalent to the following:

If $c \in \Pi$ and $\mathcal{R} \nvdash c \Rightarrow$ , then there is $t \in C^\omega$ such that $\mathcal{R}_{\mathsf{tr}} \vdash c \Rightarrow_{\mathsf{tr}} t$.

Then, the proof follows from Lemma 2. We define $\mathcal{S} = \{c \mid c{\in}\Pi \text{ and } \mathcal{R} \nvdash c \Rightarrow\}$, and show that, for all $c \in \mathcal{S}$, there are $\rho \equiv \mathsf{rule}(j_1 \dots j_n,\ j_{n+1},\ c)$ and $k \in 1..n+1$ such that, for all $h < k$, $\mathcal{R} \vdash j_h$, and $C(j_k) \in \mathcal{S}$.

Consider $c \in \mathcal{S}$, then, by **S4**, there is $\rho \equiv \mathsf{rule}(j_1 \dots j_n,\ j_{n+1},\ c)$. By definition of $\mathcal{S}$, we have $\mathcal{R} \nvdash c \Rightarrow$ , hence there exists a (first) $k \in 1..n+1$ such that $\mathcal{R} \nvdash j_k$, since, otherwise, we would have $\mathcal{R} \vdash c \Rightarrow R(j_{n+1})$. Then, since $k$ is the first index with such property, for all $h < k$, we have $\mathcal{R} \vdash j_h$, hence, again by condition **S4**, we have that $\mathcal{R} \nvdash C(j_k) \Rightarrow$ . Finally, since for all $h < k$ we have $\mathcal{R} \vdash j_h$, by Prop. 1, we get $C(j_k) \in \Pi$, hence $C(j_k) \in \mathcal{S}$, as needed.

### 5 Examples

Sect. 5.1 explains in detail how a typical soundness proof can be rephrased in terms of our technique, by reasoning directly on big-step rules. Sect. 5.2 shows a case where this is advantageous, since the property to be checked is *not preserved* by intermediate computation steps, whereas it holds for the final result. Sect. 5.3 considers a more sophisticated type system, with intersection and union types. Finally, Sect. 5.4 shows another example where subject reduction is not preserved, whereas soundness can be proved with our technique. This example is intended as a preliminary step towards a more challenging case.

#### 5.1 Simply-typed λ-calculus with recursive types

As a first example, we take the λ-calculus with natural constants, successor, and choice used in Sect. 2 (Fig. 1). We consider a standard simply-typed version with recursive types, obtained by interpreting the production in Fig. 4 coinductively. Introducing recursive types makes the calculus non-normalising and permits to write interesting programs such as $\Omega$ (see Sect. 3.1).

The typing rules are recalled in Fig. 4. Type environments, written $\Gamma$, are finite maps from variables to types, and $\Gamma\{T/x\}$ denotes the map which returns $T$ on $x$ and coincides with $\Gamma$ elsewhere. We write $\vdash e : T$ for $\emptyset \vdash e : T$.

Let $\mathcal{R}_1$ be the big-step semantics defined in Fig. 1, and let $\Pi 1_T(e)$ hold if $\vdash e : T$, for $T$ defined in Fig. 4. To prove the three conditions **S1**, **S2** and **S3** of

$$T ::= \texttt{Nat} \mid T_1 \rightarrow T_2 \text{ type}$$

$$(\textsc{t-var})\ \frac{}{\Gamma \vdash x : T}\ \Gamma(x) = T \qquad (\textsc{t-const})\ \frac{}{\Gamma \vdash n : \texttt{Nat}}$$

$$(\textsc{t-abs})\ \frac{\Gamma\{T'/x\} \vdash e : T}{\Gamma \vdash \lambda x.e : T' \rightarrow T} \qquad (\textsc{t-app})\ \frac{\Gamma \vdash e_1 : T' \rightarrow T \quad \Gamma \vdash e_2 : T'}{\Gamma \vdash e_1\ e_2 : T}$$

$$(\textsc{t-succ})\ \frac{\Gamma \vdash e : \texttt{Nat}}{\Gamma \vdash \texttt{succ}\ e : \texttt{Nat}} \qquad (\textsc{t-choice})\ \frac{\Gamma \vdash e_1 : T \quad \Gamma \vdash e_2 : T}{\Gamma \vdash e_1 \oplus e_2 : T}$$

**Fig. 4.** $\lambda$-calculus: type system

Sect. 4.2, we need lemmas of inversion, substitution and canonical forms, as in the standard technique.

**Lemma 3 (Inversion).**

1. *If* $\Gamma \vdash x : T$*, then* $\Gamma(x) = T$*.*
2. *If* $\Gamma \vdash n : T$*, then* $T = \texttt{Nat}$*.*
3. *If* $\Gamma \vdash \lambda x.e : T$*, then* $T = T_1 \rightarrow T_2$ *and* $\Gamma\{T_1/x\} \vdash e : T_2$*.*
4. *If* $\Gamma \vdash e_1\ e_2 : T$*, then* $\Gamma \vdash e_1 : T' \rightarrow T$*, and* $\Gamma \vdash e_2 : T'$*.*
5. *If* $\Gamma \vdash \texttt{succ}\ e : T$*, then* $T = \texttt{Nat}$ *and* $\Gamma \vdash e : \texttt{Nat}$*.*
6. *If* $\Gamma \vdash e_1 \oplus e_2 : T$*, then* $\Gamma \vdash e_i : T$ *with* $i \in 1, 2$*.*

**Lemma 4 (Substitution).** *If* $\Gamma\{T'/x\} \vdash e : T$ *and* $\Gamma \vdash e' : T'$*, then* $\Gamma \vdash e[e'/x] : T$*.*

**Lemma 5 (Canonical Forms).**

1. *If* $\vdash v : T' \rightarrow T$*, then* $v = \lambda x.e$*.*
2. *If* $\vdash v : \texttt{Nat}$*, then* $v = n$*.*

**Theorem 5 (Soundness).** *The big-step semantics* $\mathcal{R}_1$ *and the indexed predicate* $\Pi 1$ *satisfy the conditions* **S1**, **S2** *and* **S3** *of Sect. 4.2.*

Since the aim of this first example is to illustrate the proof technique, we provide a proof where we explain the reasoning in detail.

*Proof of **S1**.* We should prove this condition for each (instantiation of meta-)rule. (app): Assume that $\vdash e_1\ e_2 : T$ holds. We have to find types for the premises, notably $T$ for the last one. We proceed as follows:

1. First premise: by Lemma 3 (4), $\vdash e_1 : T' \rightarrow T$.
2. Second premise: again by Lemma 3 (4), $\vdash e_2 : T'$ (without needing the assumption $\vdash \lambda x.e : T' \rightarrow T$).
3. Third premise: $\vdash e[v_2/x] : T$ should hold (assuming $\vdash \lambda x.e : T' \rightarrow T$, $\vdash v_2 : T'$). Since $\vdash \lambda x.e : T' \rightarrow T$, by Lemma 3 (3) we have $x{:}T' \vdash e : T$, so by Lemma 4 and $\vdash v_2 : T'$ we have $\vdash e[v_2/x] : T$.

(SUCC): This rule has an implicit continuation $n+1 \Rightarrow n+1$. Assume that $\vdash \texttt{succ}\, e : T$ holds. By Lemma 3 (5), $T = \texttt{Nat}$, and $\vdash e : \texttt{Nat}$, hence we find Nat as type for the first premise. Moreover, $\vdash n+1 : \texttt{Nat}$ holds by rule (T-CONST).
(CHOICE): Assume that $\vdash e_1 \oplus e_2 : T$ holds. By Lemma 3 (6), we have $\vdash e_i : T$, with $i \in 1, 2$. Hence we find $T$ as type for the premise.

*Proof of **S2**.* We should prove that, for each non-result configuration (here, expression $e$ which is not a value) such that $\vdash e : T$ holds for some $T$, there is a rule with this configuration in the consequence. The expression $e$ cannot be a variable, since a variable cannot be typed in the empty environment. Application, successor and choice appear as consequence in the reduction rules.

*Proof of* **S3**. We should prove this condition for each (instantiation of meta-)rule.
(APP): Assuming $\vdash e_1\, e_2 : T$, again by Lemma 3 (4) we get $\Gamma \vdash e_1 : T' \to T$.

1. First premise: if $e1 \Rightarrow v$ is derivable, then there should be a rule with $e_1\, e_2$ in the consequence and $e1 \Rightarrow v$ as first premise. Since we proved **S1**, by preservation (Lemma 1) $\vdash v : T' \to T$ holds. Then, by Lemma 5 (1), $v$ has shape $\lambda x.e$, hence the required rule exists. As noted at page 178, in practice checking **S3** for a (meta-)rule amounts to show that (sub)configurations in the premises evaluate to results which have the required shape (to be a $\lambda$-abstraction in this case).
2. Second premise: if $e_1 \Rightarrow \lambda x.e$, and $e2 \Rightarrow v_2$, then there should be a rule with $e_1\, e_2$ in the consequence and $e_1 \Rightarrow \lambda x.e$, $e2 \Rightarrow v$ as first two premises. This is trivial since the meta-variable $v_2$ can be freely instantiated in the meta-rule.

(SUCC): Assuming $\vdash \texttt{succ}\, e : T$, again by Lemma 3 (5) we get $\vdash e : \texttt{Nat}$. If $e \Rightarrow v$ is derivable, there should be a rule with $\texttt{succ}\, e$ in the consequence and $e \Rightarrow v$ as first premise. Indeed, by preservation (Lemma 1) and Lemma 5 (2), $v$ has shape $n$. For the second premise, if $n+1 \Rightarrow v$ is derivable, then $v$ is necessarily $n+1$.
(CHOICE): Trivial since the meta-variable $v$ can be freely instantiated.

An interesting remark is that, differently from the standard approach, there is *no induction* in the proof: everything is *by cases*. This is a consequence of the fact that, as discussed in Sect. 4.2, the three conditions are *local*, that is, they are conditions on single rules. Induction is "hidden" in the proof that those three conditions are sufficient to ensure soundness.

If we drop in Fig. 1 rule (SUCC), then condition **S2** fails, since there is no longer a rule for the well-typed non-result configuration $\texttt{succ}\, n$. If we add the (FOOL) rule $\vdash 0\, 0 : \texttt{Nat}$, then condition **S3** fails for rule (APP), since $0 \Rightarrow 0$ is derivable, but there is no rule with $0\, 0$ in the conclusion and $0 \Rightarrow 0$ as first premise.

#### 5.2 MiniFJ&λ

In this example, the language is a subset of FJ&$\lambda$ [12], a calculus extending Featherweight Java (FJ) with $\lambda$-abstractions and intersection types, introduced in Java 8. To keep the example small, we do not consider intersections and focus on one key typing feature: $\lambda$-abstractions can only be typed when occurring in a context requiring a given type (called the *target type*). In a small-step semantics, this poses a problem: reduction can move $\lambda$-abstractions into arbitrary contexts, leading to intermediate terms which would be ill-typed. To maintain subject reduction, in [12] $\lambda$-abstractions are decorated with their initial target type. In a big-step semantics, there is no need of intermediate terms and annotations.

The syntax is given in the first part of Fig. 5. We assume sets of *variables* $x$, *class names* $\mathsf{C}$, *interface names* $\mathsf{I}$, $\mathsf{J}$, *field names* $\mathsf{f}$, and *method names* $\mathsf{m}$. Interfaces which have *exactly* one method (dubbed *functional interfaces*) can be used as target types. Expressions are those of FJ, plus $\lambda$-abstractions, and types are class and interface names. In $\lambda xs.e$ we assume that $xs$ is not empty and $e$ is not a $\lambda$-abstraction. For simplicity, we only consider *upcasts*, which have no runtime effect, but are important to allow the programmer to use $\lambda$-abstractions, as exemplified in discussing typing rules.

To be concise, the class table is abstractly modelled as follows:

- $\mathsf{fields}(\mathsf{C})$ gives the sequence of field declarations $T_1\,\mathsf{f}_1\,;..T_n\,\mathsf{f}_n\,;$ for class $\mathsf{C}$
- $\mathsf{mtype}(T,\mathsf{m})$ gives, for each method $\mathsf{m}$ in class or interface $T$, the pair $T_1 \ldots T_n \to T'$ consisting of the parameter types and return type
- $\mathsf{mbody}(\mathsf{C},\mathsf{m})$ gives, for each method $\mathsf{m}$ in class $\mathsf{C}$, the pair $\langle x_1 \ldots x_n,\ e\rangle$ consisting of the parameters and body
- $<:$ is the reflexive and transitive closure of the union of the $\mathsf{extends}$ and $\mathsf{implements}$ relations
- $!\mathsf{mtype}(\mathsf{I})$ gives, for each *functional* interface $\mathsf{I}$, $\mathsf{mtype}(\mathsf{I},\mathsf{m})$, where $\mathsf{m}$ is the only method of $\mathsf{I}$.

The big-step semantics is given in the last part of Fig. 5. MiniFJ&$\lambda$ shows an example of instantiation of the framework where configurations include an auxiliary structure, rather than being just language terms. In this case, the structure is an *environment* $\mathrm{E}$ (a finite map from variables to values) modelling the current stack frame. Results are values, which are either *objects*, of shape $[vs]^{\mathsf{C}}$, or $\lambda$-abstractions.

Rules for FJ constructs are straightforward. Note that, since we only consider upcasts, casts have no runtime effect. Indeed, they are guaranteed to succeed on well-typed expressions. Rule (λ-invk) shows that, when the receiver of a method is a $\lambda$-abstraction, the method name is not significant at runtime, and the effect is that the body of the function is evaluated as in the usual application.

The type system is given in Fig. 6. Method bodies are expected to be well-typed with respect to method types. Formally, $\mathsf{mbody}(\mathsf{C},\mathsf{m})$ and $\mathsf{mtype}(\mathsf{C},\mathsf{m})$ are either both defined or both undefined: in the first case $\mathsf{mbody}(\mathsf{C},\mathsf{m}) = \langle x_1 \ldots x_n,\ e\rangle$, $\mathsf{mtype}(\mathsf{C},\mathsf{m}) = T_1 \ldots T_n \to T$, and $x_1{:}T_1, \ldots, x_n{:}T_n, \mathtt{this}{:}\mathsf{C} \vdash e : T$. Moreover, we assume other standard FJ constraints on the class table, such as no field hiding, no method overloading, the same parameter and return types in overriding.

Besides the standard typing features of FJ, the MiniFJ&$\lambda$ type system ensures the following.

$$
\begin{array}{lll}
e & ::= x \mid e.\mathsf{f} \mid \mathtt{new}\ \mathsf{C}(e_1,\ldots,e_n) \mid e.\mathsf{m}(e_1,\ldots,e_n) \mid \lambda xs.e \mid (T)e & \text{expression} \\
xs & ::= x_1 \ldots x_n & \text{variable list} \\
T & ::= \mathsf{C} \mid \mathsf{I} & \text{type}
\end{array}
$$

$$
\begin{array}{lll}
c & ::= \langle \mathrm{E},\ e\rangle \mid v & \text{configuration} \\
v & ::= [vs]^{\mathsf{C}} \mid \lambda xs.e & \text{result (value)} \\
vs & ::= v_1,\ldots,v_n & \text{value list}
\end{array}
$$

$$
(\text{VAR})\ \frac{}{\langle \mathrm{E},\ x\rangle \Rightarrow v}\quad \mathrm{E}(x)=v
$$

$$
(\text{FIELD-ACCESS})\ \frac{\langle \mathrm{E},\ e\rangle \Rightarrow [v_1,\ldots,v_n]^{\mathsf{C}}}{\langle \mathrm{E},\ e.\mathsf{f}_i\rangle \Rightarrow v_i}\quad \begin{array}{l}\mathsf{fields}(\mathsf{C}) = T_1\,\mathsf{f}_1;\ldots T_n\,\mathsf{f}_n; \\ i\in 1..n\end{array}
$$

$$
(\text{NEW})\ \frac{\langle \mathrm{E},\ e_i\rangle \Rightarrow v_i\quad \forall i\in 1..n}{\langle \mathrm{E},\ \mathtt{new}\ \mathsf{C}(e_1,\ldots,e_n)\rangle \Rightarrow [v_1,\ldots,v_n]^{\mathsf{C}}}
$$

$$
(\text{INVK})\ \frac{\begin{array}{l}\langle \mathrm{E},\ e_0\rangle \Rightarrow [vs]^{\mathsf{C}} \\ \langle \mathrm{E},\ e_i\rangle \Rightarrow v_i\quad \forall i\in 1..n \\ \langle x_1{:}v_1,\ldots,x_n{:}v_n,\mathtt{this}{:}[vs]^{\mathsf{C}},\ e\rangle \Rightarrow v\end{array}}{\langle \mathrm{E},\ e_0.\mathsf{m}(e_1,\ldots,e_n)\rangle \Rightarrow v}\quad \mathsf{mbody}(\mathsf{C},\mathsf{m}) = \langle x_1\ldots x_n,\ e\rangle
$$

$$
(\lambda\text{-INVK})\ \frac{\begin{array}{l}\langle \mathrm{E},\ e_0\rangle \Rightarrow \lambda xs.e \\ \langle \mathrm{E},\ e_i\rangle \Rightarrow v_i\quad \forall i\in 1..n \\ \langle x_1{:}v_1,\ldots,x_n{:}v_n,\ e\rangle \Rightarrow v\end{array}}{\langle \mathrm{E},\ e_0.\mathsf{m}(e_1,\ldots,e_n)\rangle \Rightarrow v}\qquad (\text{UPCAST})\ \frac{\langle \mathrm{E},\ e\rangle \Rightarrow v}{\langle \mathrm{E},\ (T)e\rangle \Rightarrow v}
$$

**Fig. 5.** MiniFJ&λ: syntax and big-step semantics

– A functional interface I can be assigned as type to a λ-abstraction which has the functional type of the method, see rule (T-λ).
– A λ-abstraction should have a *target type* determined by the context where the λ-abstraction occurs. More precisely, see [25] page 602, a λ-abstraction in our calculus can only occur as return expression of a method or argument of constructor, method call or cast. Then, in some contexts a λ-abstraction cannot be typed, in our calculus when occurring as receiver in field access or method invocation, hence these cases should be prevented. This is implicit in rule (T-FIELD-ACCESS), since the type of the receiver should be a class name, whereas it is explicitly forbidden in rule (T-INVK). For the same reason, a λ-abstraction cannot be the main expression to be evaluated.
– A λ-abstraction with a given target type J should have type *exactly* J: a subtype I of J is not enough. Consider, for instance, the following program:

```
interface J {}
interface I extends J { A m(A x); }
class C {
  C m(I y) { return new C().n(y); }
  C n(J y) { return new C(); }
}
```

$$\text{(T-CONF)}\ \frac{\vdash v_i : T_i \quad \forall i \in 1..n \qquad x_1{:}T'_1, \ldots, x_n{:}T'_n \vdash e : T}{\vdash \langle x_1{:}v_1, \ldots, x_n{:}v_n,\ e\rangle : T} \quad T_i <: T'_i \quad \forall i \in 1..n$$

$$\text{(T-VAR)}\ \frac{}{\Gamma \vdash x : T} \quad \Gamma(x) = T \qquad \text{(T-FIELD-ACCESS)}\ \frac{\Gamma \vdash e : \mathsf{C}}{\Gamma \vdash e.\mathsf{f} : T_i} \quad \begin{array}{l}\mathsf{fields}(\mathsf{C}) = T_1\,\mathsf{f}_1; \ldots T_n\,\mathsf{f}_n;\\ i \in 1..n\end{array}$$

$$\text{(T-NEW)}\ \frac{\Gamma \vdash e_i : T_i \quad \forall i \in 1..n}{\Gamma \vdash \mathtt{new}\ \mathsf{C}(e_1, \ldots, e_n) : \mathsf{C}} \quad \mathsf{fields}(\mathsf{C}) = T_1\,\mathsf{f}_1; \ldots T_n\,\mathsf{f}_n;$$

$$\text{(T-INVK)}\ \frac{\Gamma \vdash e_i : T_i \quad \forall i \in 0..n}{\Gamma \vdash e_0.\mathsf{m}(e_1, \ldots, e_n) : T} \quad \begin{array}{l} e_0 \text{ not of shape } \lambda xs.e\\ \mathsf{mtype}(T_0, \mathsf{m}) = T_1 \ldots T_n \to T\end{array}$$

$$\text{(T-}\lambda\text{)}\ \frac{x_1{:}T_1, \ldots, x_n{:}T_n \vdash e : T}{\Gamma \vdash \lambda xs.e : \mathsf{I}} \quad !\mathsf{mtype}(\mathsf{I}) = T_1 \ldots T_n \to T$$

$$\text{(T-UPCAST)}\ \frac{\Gamma \vdash e : T}{\Gamma \vdash (T)e : T} \qquad \text{(T-OBJECT)}\ \frac{\Gamma \vdash v_i : T'_i \quad \forall i \in 1..n}{\Gamma \vdash [v_1, \ldots, v_n]^{\mathsf{C}} : \mathsf{C}} \quad \begin{array}{l}\mathsf{fields}(\mathsf{C}) = T_1\,\mathsf{f}_1; \ldots T_n\,\mathsf{f}_n;\\ T'_i <: T_i \quad \forall i \in 1..n\end{array}$$

$$\text{(T-SUB)}\ \frac{\Gamma \vdash e : T}{\Gamma \vdash e : T'} \quad \begin{array}{l} e \text{ not of shape } \lambda xs.e\\ T <: T'\end{array}$$

**Fig. 6.** MiniFJ&λ: type system and the main expression new $\mathsf{C}().\mathsf{n}(\lambda x.x)$. Here, the λ-abstraction has target type J, which is *not* a functional interface, hence the expression is ill-typed in Java (the compiler has no functional type against which to type-check the λ-abstraction). On the other hand, in the body of method m, the parameter $y$ of type I can be passed, as usual, to method n expecting a supertype. For instance, the main expression new $\mathsf{C}().\mathsf{m}(\lambda x.x)$ is well-typed, since the λ-abstraction has target type I, and can be safely passed to method n, since it is not used as function there. To formalise this behaviour, it is forbidden to apply subsumption to λ-abstractions, see rule (T-SUB).

– However, λ-abstractions occurring as results rather than in source code (that is, in the environment and as fields of objects) are allowed to have a subtype of the required type, see the explicit side condition in rules (T-CONF) and (T-OBJECT). For instance, if C is a class with one field J f, the expression new $\mathsf{C}((\mathsf{I})\lambda x.x)$ is well-typed, whereas new $\mathsf{C}(\lambda x.x)$ is ill typed, since rule (T-SUB) cannot be applied to λ-abstractions. When the expression is evaluated, the result is $[\lambda x.x]^{\mathsf{C}}$, which is well-typed.

As mentioned at the beginning, the obvious small-step semantics would produce not typable expressions. In the above example, we get

$$\mathtt{new}\ \mathsf{C}((\mathsf{I})\lambda x.x) \longrightarrow \mathtt{new}\ \mathsf{C}(\lambda x.x) \longrightarrow [\lambda x.x]^{\mathsf{C}}$$

and new $\mathsf{C}(\lambda x.x)$ has no type, while new $\mathsf{C}((\mathsf{I})\lambda x.x)$ and $[\lambda x.x]^{\mathsf{C}}$ have type C.

We write $\Gamma \vdash e :<: T$ as short for $\Gamma \vdash e : T'$ and $T' <: T$ for some $T'$. In order to state soundness, set $\mathcal{R}_2$ the big-step semantics defined in Fig. 5, and let

$\Pi 2_T(\langle \mathrm{E},\ e\rangle)$ hold if $\vdash \langle \mathrm{E},\ e\rangle$ :<: $T$, $\Pi 2_T(v)$ if $\vdash v$ :<: $T$, for $T$ defined in Fig. 5.

**Theorem 6 (Soundness).** *The big-step semantics $\mathcal{R}_2$ and the indexed predicate $\Pi 2$ satisfy the conditions* **S1**, **S2** *and* **S3** *of Sect. 4.2.*

#### 5.3 Intersection and union types

We enrich the type system of Fig. 4 by adding intersection and union type constructors and the corresponding typing rules, see Fig. 7. As usual we require an infinite number of arrows in each infinite path for the trees representing types. Intersection types for the λ-calculus have been widely studied [11]. Union types naturally model conditionals [26] and non-deterministic choice [22].

$$T ::= \mathtt{Nat} \mid T_1 \rightarrow T_2 \mid T_1 \wedge T_2 \mid T_1 \vee T_2 \quad \text{type}$$

$$(\wedge\,\mathrm{I})\ \frac{\Gamma \vdash e : T \quad \Gamma \vdash e : S}{\Gamma \vdash e : T \wedge S} \qquad (\wedge\,\mathrm{E})\ \frac{\Gamma \vdash e : T \wedge S}{\Gamma \vdash e : T} \qquad (\wedge\,\mathrm{E})\ \frac{\Gamma \vdash e : T \wedge S}{\Gamma \vdash e : S}$$

$$(\vee\,\mathrm{I})\ \frac{\Gamma \vdash e : T}{\Gamma \vdash e : T \vee S} \qquad (\vee\,\mathrm{I})\ \frac{\Gamma \vdash e : S}{\Gamma \vdash e : T \vee S}$$

**Fig. 7.** Intersection and union types: syntax and typing rules

The typing rules for the introduction and the elimination of intersection and union are standard, except for the absence of the union elimination rule:

$$(\vee E)\ \frac{\Gamma\{T/x\} \vdash e : V \quad \Gamma\{S/x\} \vdash e : V \quad \Gamma \vdash e' : T \vee S}{\Gamma \vdash e[e'/x] : V}$$

As a matter of fact rule $(\vee E)$ is unsound for $\oplus$. For example, let split the type `Nat` into `Even` and `Odd` and add the expected typings for natural numbers. The prefix addition $+$ has type

$$(\mathtt{Even} \rightarrow \mathtt{Even} \rightarrow \mathtt{Even}) \wedge (\mathtt{Odd} \rightarrow \mathtt{Odd} \rightarrow \mathtt{Even})$$

and we derive

$$\frac{x{:}\mathtt{Even} \vdash +\,x\,x{:}\mathtt{Even} \quad x{:}\mathtt{Odd} \vdash +\,x\,x{:}\mathtt{Even} \quad \dfrac{\dfrac{\vdash 1 : \mathtt{Odd}}{\vdash 1 : \mathtt{Even} \vee \mathtt{Odd}}(\vee\,\mathrm{I}) \quad \dfrac{\vdash 2 : \mathtt{Even}}{\vdash 2 : \mathtt{Even} \vee \mathtt{Odd}}(\vee\,\mathrm{I})}{\vdash (1 \oplus 2) : \mathtt{Even} \vee \mathtt{Odd}}(\oplus)}{\vdash +(1 \oplus 2)(1 \oplus 2) : \mathtt{Even}}(\vee\,\mathrm{E})$$

We cannot assign the type `Even` to 3, which is a possible result, so strong soundness is lost. In the small-step approach, we cannot assign `Even` to the intermediate term $+\,1\,2$, so subject reduction fails. In the big-step approach, there is no such intermediate term; however, condition **S1** fails for the reduction rule for $+$. Indeed, considering the following instantiation of the rule:

$$(+)\ \frac{1 \oplus 2 \Rightarrow 1 \quad 1 \oplus 2 \Rightarrow 2 \quad 3 \Rightarrow 3}{+(1 \oplus 2)(1 \oplus 2) \Rightarrow 3}$$

and the type `Even` for the consequence, we cannot assign this type to the (configuration in) last premise (continuation).

Intersection types allow to derive meaningful types also for expressions containing variables applied to themselves, for example we can derive

$$\vdash \lambda x.x\,x : (T \rightarrow S) \wedge T \rightarrow S$$

With union types all non-deterministic choices between typable expressions can be typed too, since we can derive $\Gamma \vdash e_1 \oplus e_2 : T_1 \vee T_2$ from $\Gamma \vdash e_1 : T_1$ and $\Gamma \vdash e_2 : T_2$.

In order to state soundness, let $\Pi 3_T(e)$ be $\vdash e : T$, for $T$ defined in Fig. 7.

**Theorem 7 (Soundness).** *The big-step semantics* $\mathcal{R}_1$ *and the indexed predicate* $\Pi 3$ *satisfy the conditions* **S1**, **S2** *and* **S3** *of Sect. 4.2.*

#### 5.4 MiniFJ&O

A well-known example in which proving soundness with respect to small-step semantics is extremely challenging is the standard type system with intersection and union types [10] w.r.t. the pure $\lambda$-calculus with full reduction. Indeed, the standard subject reduction technique fails[5], since, for instance, we can derive the type $(T \rightarrow T \rightarrow V) \wedge (S \rightarrow S \rightarrow V) \rightarrow (U \rightarrow T \vee S) \rightarrow U \rightarrow V$ for both $\lambda x.\lambda y.\lambda z.x((\lambda t.t)(y\,z))((\lambda t.t)(y\,z))$ and $\lambda x.\lambda y.\lambda z.x(y\,z)(y\,z)$, but the intermediate expressions $\lambda x.\lambda y.\lambda z.x((\lambda t.t)(y\,z))(y\,z)$ and $\lambda x.\lambda y.\lambda z.x(y\,z)((\lambda t.t)(y\,z))$ do not have this type.

As the example shows, the key problem is that rule $(\vee E)$ can be applied to expression $e$ where the same subexpression $e'$ occurs more than once. In the non-deterministic case, as shown by the example in the previous section, this is unsound, since $e'$ can reduce to different values. In the deterministic case, instead, this is sound, but cannot be proved by subject reduction. Since using big-step semantics there are no intermediate steps to be typed, our approach seems very promising to investigate an alternative proof of soundness. Whereas we leave this challenging problem to future work, here as first step we describe a (hypothetical) calculus with a much simpler version of the problematic feature.

The calculus is a variant of FJ [27] with intersection and union types. Methods have intersection types with the same return type and different parameter types, modelling a form of *overloading*. Union types enhance typability of conditionals. The more interesting feature is the possibility of replacing an arbitrary number of parameters with the same expression having an union type. We dub this calculus MiniFJ&O.

Fig. 8 gives the syntax, big-step semantics and typing rules of MiniFJ&O. We omit the standard big-step rule for conditional, and typing rules for boolean

[5] For this reason, in [10] soundness is proved by an ad-hoc technique, that is, by considering parallel reduction and an equivalent type system à la Gentzen, which enjoys the cut elimination property.

$$\begin{array}{llr} e & ::= x \mid v \mid e.\mathsf{f} \mid e.\mathsf{m}(e_1,\ldots,e_n) \mid \mathsf{if}\ e\ \mathsf{then}\ e_1\ \mathsf{else}\ e_2 & \text{expression} \\ v & ::= \mathtt{new}\ \mathsf{C}(v_1,\ldots,v_n) \mid \mathtt{true} \mid \mathtt{false} & \text{value} \\ T & ::= \mathsf{C} \mid \mathtt{Bool} \mid \bigvee_{1\le i\le n} T_i & \text{expression type} \\ MT & ::= \bigwedge_{1\le i\le m}(\mathsf{C}_1^{(i)}\ldots\mathsf{C}_n^{(i)} \to \mathsf{D}) & \text{method type} \end{array}$$

$$\text{(FIELD-ACCESS)}\ \frac{e \Rightarrow \mathtt{new}\ \mathsf{C}(v_1,\ldots,v_n)}{e.\mathsf{f}_i \Rightarrow v_i}\ \begin{array}{l}\mathsf{fields}(\mathsf{C}) = T_1\,\mathsf{f}_1;\ldots T_n\,\mathsf{f}_n; \\ i \in 1..n\end{array}$$

$$\text{(NEW)}\ \frac{e_i \Rightarrow v_i \quad \forall i \in 1..n}{\mathtt{new}\ \mathsf{C}(e_1,\ldots,e_n) \Rightarrow \mathtt{new}\ \mathsf{C}(v_1,\ldots,v_n)}$$

$$\text{(INVK)}\ \frac{\begin{array}{l} e_0 \Rightarrow \mathtt{new}\ \mathsf{C}(vs') \\ e_i \Rightarrow v_i \quad \forall i \in 1..n \\ e[v_1/x_1]\ldots[v_n/x_n][\mathtt{new}\ \mathsf{C}(vs')/\mathtt{this}] \Rightarrow v \end{array}}{e_0.\mathsf{m}(e_1,\ldots,e_n) \Rightarrow v}\ \mathsf{mbody}(\mathsf{C},\mathsf{m}) = \langle x_1\ldots x_n,\ e\rangle$$

$$\text{(T-VAR)}\ \frac{}{\Gamma \vdash x : T}\ \Gamma(x) = T \qquad \text{(T-FIELD-ACCESS)}\ \frac{\Gamma \vdash e : \mathsf{C}}{\Gamma \vdash e.\mathsf{f}_i : \mathsf{C}_i}\ \begin{array}{l}\mathsf{fields}(\mathsf{C}) = T_1\,\mathsf{f}_1;\ldots T_n\,\mathsf{f}_n; \\ i \in 1..n\end{array}$$

$$\text{(T-NEW)}\ \frac{\Gamma \vdash e_i : \mathsf{C}_i \quad \forall i \in 1..n}{\Gamma \vdash \mathtt{new}\ \mathsf{C}(e_1,\ldots,e_n) : \mathsf{C}}\ \mathsf{fields}(\mathsf{C}) = T_1\,\mathsf{f}_1;\ldots T_n\,\mathsf{f}_n;$$

$$\text{(T-INVK)}\ \frac{\Gamma \vdash e_i : \mathsf{C}_i \quad \forall i \in 0..n \quad \Gamma \vdash e : \bigvee_{1\le i\le m} \mathsf{D}_i}{\Gamma \vdash e_0.\mathsf{m}(e_1,\ldots,e_n,\underbrace{e,\ldots,e}_{p}) : \mathsf{C}}\ \begin{array}{l}\mathsf{mtype}(\mathsf{C}_0,\mathsf{m}) <: \\ \bigwedge_{1\le i\le m}(\mathsf{C}_1\ldots\mathsf{C}_n\,\underbrace{\mathsf{D}_i\ldots\mathsf{D}_i}_{p} \to \mathsf{C})\end{array}$$

$$\text{(T-IF)}\ \frac{\Gamma \vdash e : \mathtt{Bool} \quad \Gamma \vdash e_1 : T \quad \Gamma \vdash e_2 : T}{\Gamma \vdash \mathsf{if}\ e\ \mathsf{then}\ e_1\ \mathsf{else}\ e_2 : T} \qquad \text{(T-SUB)}\ \frac{\Gamma \vdash e : T}{\Gamma \vdash e : T'}\ T <: T'$$

**Fig. 8.** MiniFJ&O: syntax, big-step semantics and type system constants. The subtyping relation <: is the reflexive and transitive closure of the union of the extends relation and the standard rules for union:

$$T_1 <: T_1 \vee T_2 \qquad T_1 <: T_2 \vee T_1$$

On the other hand, *method types* (results of the mtype function) are now *intersection types*, and the subtyping relation on them is the reflexive and transitive closure of the standard rules for intersection:

$$MT_1 \wedge MT_2 <: MT_1 \qquad MT_1 \wedge MT_2 <: MT_2$$

The functions fields and mbody are defined as for MiniFJ&λ.
Instead $\mathsf{mtype}(\mathsf{C},\mathsf{m})$ gives, for each method m in class C, an intersection type. We assume $\mathsf{mbody}(\mathsf{C},\mathsf{m})$ and $\mathsf{mtype}(\mathsf{C},\mathsf{m})$ either both defined or both undefined: in the first case $\mathsf{mbody}(\mathsf{C},\mathsf{m}) = \langle x_1\ldots x_n,\ e\rangle$, $\mathsf{mtype}(\mathsf{C},\mathsf{m}) = \bigwedge_{1\le i\le m}(\mathsf{C}_1^{(i)}\ldots\mathsf{C}_n^{(i)} \to \mathsf{D})$, and $x_1{:}\mathsf{C}_1^{(i)},\ldots,x_n{:}\mathsf{C}_n^{(i)},\mathtt{this}{:}\mathsf{C} \vdash e : \mathsf{D}$ for $i \in 1..m$.

Clearly rule (T-INVK) is inspired by rule ($\vee E$), but the restriction to method calls endows a standard inversion lemma. The subtyping in this rule allows to choose the types for the method best fitting the types of the arguments. Not surprisingly, subject reduction fails for the expected small-step semantics. For example, let class $\mathsf{C}$ have a field $\mathsf{point}$ which contains cartesian coordinates and class $\mathsf{D}$ have a field $\mathsf{point}$ which contains polar coordinates. The method $\mathsf{eq}$ takes two objects and compares their $\mathsf{point}$ fields returning a boolean value. A type for this method is $(\mathsf{C}\,\mathsf{C} \to \mathtt{Bool}) \wedge (\mathsf{D}\,\mathsf{D} \to \mathtt{Bool})$ and we can type $\mathsf{eq}(e, e)$, where

$$e = \mathsf{if}\ \mathtt{false}\ \mathsf{then}\ \mathtt{new}\ \mathsf{C}(\ldots)\ \mathsf{else}\ \mathtt{new}\ \mathsf{D}(\ldots)$$

In fact $e$ has type $\mathsf{C} \vee \mathsf{D}$. Notice that in a standard small-step semantics

$$\mathsf{eq}(e, e) \longrightarrow \mathsf{eq}(\mathtt{new}\ \mathsf{D}(\ldots), \mathsf{if}\ \mathtt{false}\ \mathsf{then}\ \mathtt{new}\ \mathsf{C}(\ldots)\ \mathsf{else}\ \mathtt{new}\ \mathsf{D}(\ldots))$$

and this last expression cannot be typed.

In order to state soundness, let $\mathcal{R}_4$ be the big-step semantics defined in Fig. 8, and let $\Pi 4_T(e)$ hold if $\vdash e : T$, for $T$ defined in Fig. 8.

**Theorem 8 (Soundness).** *The big-step semantics $\mathcal{R}_4$ and the indexed predicate $\Pi 4$ satisfy the conditions* **S1**, **S2** *and* **S3** *of Sect. 4.2.*

### 6 The partial evaluation construction

In this section, our aim is to provide a *formal* justification that the constructions in Sect. 3 are correct. For instance, for the $\mathsf{wrong}$ semantics we would like to be sure that *all* the cases are covered. To this end, we define a *third construction*, dubbed PEV for "partial evaluation", which makes explicit the *computations* of a big-step semantics, intended as the sequences of execution steps of the naturally associated evaluation algorithm. Formally, we obtain a reduction relation on approximated proof trees, so non-termination and stuck computation are distinguished, and both soundness-must and soundness-may can be expressed.

To this end, first of all we introduce a special result ?, so that a judgment $c \Rightarrow ?$ (called *incomplete*, whereas a judgment in $\mathcal{R}$ is *complete*) means that the evaluation of $c$ is not completed yet. Analogously to the previous constructions, we define an augmented set of rules $\mathcal{R}_?$ for the judgment extended with ?:

**? introduction rules** These rules derive ? whenever a rule is partially applied: for each rule $\rho \equiv \mathsf{rule}(j_1 \ldots j_n,\ j_{n+1},\ c)$ in $\mathcal{R}$, index $i \in 1..n+1$, and result $r \in R$, we define the rule $\mathsf{intro}_?(\rho,\ i,\ r)$ as

$$\frac{j_1 \quad \ldots \quad j_{i-1} \quad C(j_i) \Rightarrow r}{c \Rightarrow ?}$$

We also add an axiom $\dfrac{}{c \Rightarrow ?}$ for each configuration $c \in C$.

**? propagation rules** These rules propagate ? analogously to those for divergence and $\mathsf{wrong}$ propagation: for each $\rho \equiv \mathsf{rule}(j_1 \ldots j_n,\ j_{n+1},\ c)$ in $\mathcal{R}$, and index $i \in 1..n+1$, we add the rule $\mathsf{prop}(\rho, i, ?)$ as follows:

$$\frac{j_1 \quad \ldots \quad j_{i-1} \quad C(j_i) \Rightarrow ?}{c \Rightarrow ?}$$

Finally, we consider the set $\mathcal{T}$ of the (finite) proof trees $\tau$ in $\mathcal{R}_?$. Each $\tau$ can be thought as a *partial proof* or *partial evaluation* of the root configuration. In particular, we say it is *complete* if it is a proof tree in $\mathcal{R}$ (that is, it only contains complete judgments), *incomplete* otherwise. We define a reduction relation $\xrightarrow{\mathcal{R}}$

$$\text{(}r_?\text{)}\,\frac{}{r \Rightarrow ?} \xrightarrow{\mathcal{R}} \text{(}r\text{)}\,\frac{}{r \Rightarrow r} \qquad \text{(}c_?\text{)}\,\frac{}{c \Rightarrow ?} \xrightarrow{\mathcal{R}} (\mathsf{prop}(\rho,1,?))\,\frac{c' \Rightarrow ?}{c \Rightarrow ?}\ \begin{array}{l} C(\rho) = c \\ C(\rho,1) = c' \end{array}$$

$$(\mathsf{intro}_?(\rho,\, i,\, r))\,\frac{\mathcal{T}_1\ \ldots\ \mathcal{T}_i}{c \Rightarrow ?} \xrightarrow{\mathcal{R}} (\rho')\,\frac{\mathcal{T}_1\ \ldots\ \mathcal{T}_i}{c \Rightarrow r}\ \begin{array}{l} \rho' \sim_i \rho \\ R(\rho', i) = r \\ \#\rho' = i \end{array}$$

$$(\mathsf{intro}_?(\rho,\, i,\, r))\,\frac{\mathcal{T}_1\ \ldots\ \mathcal{T}_i}{c \Rightarrow ?} \xrightarrow{\mathcal{R}} (\mathsf{prop}(\rho', i{+}1, ?))\,\frac{\mathcal{T}_1\ \ldots\ \mathcal{T}_i\ c' \Rightarrow ?}{c \Rightarrow ?}\ \begin{array}{l} \rho' \sim_i \rho \\ R(\rho', i) = r \\ C(\rho', i+1) = c' \end{array}$$

$$(\mathsf{prop}(\rho,i,?))\,\frac{\mathcal{T}_1\ \ldots\ \mathcal{T}_i}{c \Rightarrow ?} \xrightarrow{\mathcal{R}} (\mathsf{prop}(\rho,i,?))\,\frac{\mathcal{T}_1\ \ldots\ \mathcal{T}_{i-1}\ \mathcal{T}'_i}{c \Rightarrow ?}\ \begin{array}{l} \mathcal{T}_i \xrightarrow{\mathcal{R}} \mathcal{T}'_i \\ R_?(\mathsf{r}(\mathcal{T}'_i)) = ? \end{array}$$

$$(\mathsf{prop}(\rho,i,?))\,\frac{\mathcal{T}_1\ \ldots\ \mathcal{T}_i}{c \Rightarrow ?} \xrightarrow{\mathcal{R}} (\mathsf{intro}_?(\rho,\, i,\, r))\,\frac{\mathcal{T}_1\ \ldots\ \mathcal{T}_{i-1}\ \mathcal{T}'_i}{c \Rightarrow ?}\ \begin{array}{l} \mathcal{T}_i \xrightarrow{\mathcal{R}} \mathcal{T}'_i \\ R_?(\mathsf{r}(\mathcal{T}'_i)) = r \end{array}$$

**Fig. 9.** Reduction relation on $\mathcal{T}$

on $\mathcal{T}$ such that, starting from the initial proof tree $\frac{}{c \Rightarrow ?}$, we derive a sequence where, intuitively, at each step we detail the proof (evaluation). In this way, a sequence ending with a complete tree $\frac{\cdots}{c \Rightarrow r}$ models terminating computation, whereas an infinite sequence (tending to an infinite proof tree) models divergence, and a stuck sequence models a stuck computation.

The one-step reduction relation $\xrightarrow{\mathcal{R}}$ on $\mathcal{T}$ is inductively defined by the rules in Fig. 9. In this figure $\#\rho$ denotes the number of premises of $\rho$, and $\mathsf{r}(\tau)$ the root of $\tau$. We set $R_?(c \Rightarrow u) = u$ where $u \in R \cup \{?\}$. Finally, $\sim_i$ is the *equivalence up-to an index* of rules, introduced at the beginning of Sect. 3.2. As said above, each reduction step makes "less incomplete" the proof tree. Notably, reduction rules apply to nodes with consequence $c \Rightarrow ?$, whereas subtrees with root $c \Rightarrow r$ represent terminated evaluation. In detail:

- If the last applied rule is an axiom, and the configuration is a result $r$, then we can evaluate $r$ to itself. Otherwise, we have to find a rule $\rho$ with $c$ in the consequence and start evaluating the first premise of such rule.
- If the last applied rule is $\mathsf{intro}_?(\rho,\, i,\, r)$, then all subtrees are complete, hence, to continue the evaluation, we have to find another rule $\rho'$, having, for each $k \in 1..i$, as $k$-th premise the root of $\tau_k$. Then there are two possibilities: if there is an $i+1$-th premise, we start evaluating it, otherwise, we propagate to the conclusion the result $r$ of $\tau_i$.
- If the last applied rule is a propagation rule $\mathsf{prop}(\rho, i, ?)$, then we simply propagate the step made by $\tau_i$.

In Fig. 10 we report an example of PEV reduction.

We end by stating the three constructions to be equivalent to each other, thus providing a coherency result of the approach. In particular, first we show that PEV is conservative with respect to $\mathcal{R}$, and this ensures the three constructions are equivalent for finite computations. Then, we prove traces and $\mathsf{wrong}$

$$\frac{}{(\lambda x.x)\ n \Rightarrow ?} \xrightarrow{\mathcal{R}} \frac{\lambda x.x \Rightarrow ?}{(\lambda x.x)\ n \Rightarrow ?} \xrightarrow{\mathcal{R}} \frac{\lambda x.x \Rightarrow \lambda x.x}{(\lambda x.x)\ n \Rightarrow ?} \xrightarrow{\mathcal{R}} \frac{\lambda x.x \Rightarrow \lambda x.x \quad n \Rightarrow ?}{(\lambda x.x)\ n \Rightarrow ?}$$

$$\xrightarrow{\mathcal{R}} \frac{\lambda x.x \Rightarrow \lambda x.x \quad n \Rightarrow n}{(\lambda x.x)\ n \Rightarrow ?} \xrightarrow{\mathcal{R}} \frac{\lambda x.x \Rightarrow \lambda x.x \quad n \Rightarrow n \quad n \Rightarrow ?}{(\lambda x.x)\ n \Rightarrow ?}$$

$$\xrightarrow{\mathcal{R}} \frac{\lambda x.x \Rightarrow \lambda x.x \quad n \Rightarrow n \quad n \Rightarrow n}{(\lambda x.x)\ n \Rightarrow ?} \xrightarrow{\mathcal{R}} \frac{\lambda x.x \Rightarrow \lambda x.x \quad n \Rightarrow n \quad n \Rightarrow n}{(\lambda x.x)\ n \Rightarrow n}$$

**Fig. 10.** The evaluation in PEV of $(\lambda x.x)\ n$.

constructions to be equivalent to PEV for diverging and stuck computations, respectively, and this ensures they cover all possible cases.

**Theorem 9.** *1.* $\mathcal{R} \vdash c \Rightarrow r$ *iff* $\frac{}{c \Rightarrow ?} \xrightarrow{\mathcal{R}}{}^{\star} \tau$, *where* $\mathsf{r}(\tau) = c \Rightarrow r$.

*2.* $\mathcal{R}_{\mathsf{tr}} \vdash c \Rightarrow_{\mathsf{tr}} t$ *for some* $t \in C^{\omega}$ *iff* $\frac{}{c \Rightarrow ?} \xrightarrow{\mathcal{R}}{}^{\omega}$.

*3.* $\mathcal{R}_{\mathsf{wr}} \vdash c \Rightarrow \mathsf{wrong}$ *iff* $\frac{}{c \Rightarrow ?} \xrightarrow{\mathcal{R}}{}^{\star} \tau$, *where* $\tau$ *is stuck.*

## 7 Related work

*Modeling divergence* The issue of modelling divergence in big-step semantics dates back to [18], where a stratified approach with a separate coinductive judgment for divergence is proposed, also investigated in [30].

In [5] the authors models divergence by interpreting coinductively standard big-step rules and considering also non-well-founded values. In [17] a similar technique is exploited, by adding a special result modelling divergence. Flag-based big-step semantics [36] captures divergence by interpreting the same semantic rules both inductively and coinductively. In all these approaches, spurious judgements can be derived for diverging computations.

Other proposals [32,3] are inspired by the notion of definitional interpreter [37], where a counter limits the number of steps of a computation. Thus, divergence can be modelled on top of an inductive judgement: a program diverges if the timeout is raised for any value of the counter, hence it is not directly modelled in the definition. Instead, [20] provides a way to directly model divergence using definitional interpreters, relying on the coinductive partiality monad [16].

The trace semantics in Sect. 3.1 has been inspired by [29]. Divergence propagation rules are very similar to those used in [8,9] to define a big-step judgment which directly includes divergence as result. However, this direct definition relies on a non-standard notion of inference system, allowing *corules* [7,19], whereas for the trace semantics presented in this work standard coinduction is enough, since all rules are *productive*, that is, they always add an element to the trace.

Differently from all the previously cited papers which consider specific examples, the work [2] shares with us the aim of providing a *generic construction* to model non-termination, basing on an arbitrary big-step semantics. Ager considers a class of big-step semantics identified by a specific shape of rules, and defines, in a small-step style, a proof-search algorithm which follows the big-step rules; in this way, converging, diverging and stuck computations are distinguished. This approach is somehow similar to our PEV semantics, even tough the transition system we propose is directly defined on proof trees.

There is an extensive body of work on coalgebraic techniques, where the difference between semantics can be simply expressed by a change of functor. In this paper we take a set-theoretic approach, simple and accessible to a large audience. Furthermore, as far as we know [38], coalgebras abstract several kinds of transition systems, thus being more similar to a small-step approach. In our understanding, the coalgebra models a single computation step with possible effects, and from this it is possible to derive a unique morphism into the final coalgebra modelling the "whole" semantics. Our trace semantics, being big-step, seems to roughly correspond to directly get this whole semantics. In other words, we do not have a coalgebra structure on configurations.

*Proving soundness* As we have discussed, also proving (type) soundness with respect to a big-step semantics is a challenging task, and some approaches have been proposed in the literature. In [24], to show soundness of large steps semantics, they prove a coverage lemma, which ensures that the rules cover all cases, including error situations. In [30] the authors prove a soundness property similar to Theorem 4, but by using a separate judgment to represent divergence, thus avoiding using traces. In [5] there is a proof of soundness of a coinductive type system with respect to a coinductive big-step semantics for a Java-like language, defining a relation between derivations in the type system and in the big-step semantics. In [8] there is a proof principle, used to show type soundness with respect to a big-step semantics defined by an inference system with corules [7]. In [4] the proof of type soundness of a calculus formalising path-dependent types relies on a big-step semantics, while in [3] soundness is shown for the polymorphic type systems $F_{<:}$, and for the DOT calculus, using definitional interpreters to model the semantics. In both cases they extend the original semantics adding error and timeout, and adopt inductive proof strategies, as in [39]. A similar approach is followed by [32] to show type soundness of the Core ML language.

Also [6] proposes an inductive proof of type soundness for the big-step semantics of a Java-like language, but relying on a notion of approximation of infinite derivation in the big-step semantics.

Pretty big-step semantics [17] aims at providing an efficient representation of big-step semantics, so that it can be easily extended without duplication of meta-rules. In order to define and prove soundness, they propose a generic error rule based on a *progress judgment*, whose definition can be easily derived manually from the set of evaluation rules. This is partly similar to our wrong extension, with two main differences. First, by factorising rules, they introduce intermediate steps as in small-step semantics, hence there are similar problems when intermediate steps are ill-typed (as in Sect. 5.2, Sect. 5.4). Second, wrong introduction is handled by the progress judgment, that is, at the level of sideconditions. Moreover, in [13] there is a formalisation of the pretty-big-step rules for performing a generic reasoning on big-step semantics by using abstract interpretation. However, the authors say that they interpret rules inductively, hence non-terminating computations are not modelled.

Finally, some (but not all) infinite trees of our trace semantics can be seen as cyclic proof trees, see end of Sect. 3.1. Proof systems supporting cyclic proofs can be found, e.g., in [14,15] for classical first order logic with inductive definitions.

### 8 Conclusion and future work

The most important contribution is a general approach for reasoning on soundness with respect to a big-step operational semantics. Conditions can be proven by a case analysis on the semantic (meta-)rules avoiding small-step-style intermediate configurations. This can be crucial since there are calculi where the property to be checked is *not preserved* by such intermediate configurations, whereas it holds for the final result, as illustrated in Sect. 5.

In future work, we plan to use the meta-theory in Sect. 2 as basis to investigate yet other constructions, notably the approach relying on corules [8,9], and that, adding a counter, based on timeout [32,3].

We also plan to compare our proof technique for proving soundness with the standard one for small-step semantics: if a predicate satisfies progress and subject reduction with respect to a small-step semantics, does it satisfy our soundness conditions with respect to an equivalent big-step semantics? To formally prove such a statement, the first step will be to express equivalence between small-step and big-step semantics. On the other hand, the converse does not hold, as shown by the examples in Sect. 5.2 and Sect. 5.4.

For what concerns significant applications, we plan to use the approach to prove soundness for the $\lambda$-calculus with full reduction and intersection/union types [10]. The interest of this example lies in the failure of the subject reduction, as discussed in Sect. 5.4. In another direction, we want to enhance MiniFJ&O with $\lambda$-abstractions and allowing everywhere intersection and union types [23]. This will extend typability of shared expressions. We plan to apply our approach to the big-step semantics of the statically typed virtual classes calculus developed in [24], discussing also the non terminating computations not considered there.

With regard to proofs, that are mainly omitted here, and can be found in the extended version at http://arxiv.org/abs/2002.08738, we plan to investigate if we can simplify them by means of enhanced conductive techniques.

As a proof-of-concept, we provided a mechanisation[6] in Agda of Lemma 1. The mechanisations of the other proofs is similar. However, as future work, we think it would be more interesting to provide a software for writing big-step definitions and for checking that the soundness conditions hold.

*Acknowledgments* The authors are grateful to the referees: the paper strongly improved thanks to their useful suggestions and remarks.

[6] Available at https://github.com/fdgn/soundness-big-step-semantics.
